# A meaningful MESS (Medical Education Scholarship Support)

**DOI:** 10.3402/meo.v21.32458

**Published:** 2016-07-29

**Authors:** Shari A. Whicker, Deborah L. Engle, Saumil Chudgar, Stephen DeMeo, Sarah M. Bean, Aditee P. Narayan, Colleen O'Connor Grochowski, Alisa Nagler

**Affiliations:** 1 Department of Pediatrics, Virginia Tech Carilion School of Medicine, Roanoke, VA, USA; 2 Office of Curricular Affairs, Duke University School of Medicine, Durham, NC, USA; 3 Department of Medicine, Duke University School of Medicine, Durham, NC, USA; 4 Department of Pediatrics, WakeMed Health & Hospitals, Raleigh, NC, USA; 5 Division of Pathology Clinical Services, Department of Pathology, Duke University Medical Center, Durham, NC, USA; 6 Department of Pediatrics, Duke University Medical Center, Durham, NC, USA; 7 Division of Education, American College of Surgeons, Chicago, IL, USA

**Keywords:** health professions education research, support community, fostering scholarship

## Abstract

**Background:**

Graduate medical education faculty bear the responsibility of demonstrating active research and scholarship; however, faculty who choose education-focused careers may face unique obstacles related to the lack of promotion tracks, funding, career options, and research opportunities. Our objective was to address education research and scholarship barriers by providing a collaborative peer-mentoring environment and improve the production of research and scholarly outputs.

**Methods:**

We describe a Medical Education Scholarship Support (MESS) group created in 2013. MESS is an interprofessional, multidisciplinary peer-mentoring education research community that now spans multiple institutions. This group meets monthly to address education research and scholarship challenges. Through this process, we develop new knowledge, research, and scholarly products, in addition to meaningful collaborations.

**Results:**

MESS originated with eight founding members, all of whom still actively participate. MESS has proven to be a sustainable unfunded local community of practice, encouraging faculty to pursue health professions education (HPE) careers and fostering scholarship. We have met our original objectives that involved maintaining 100% participant retention; developing increased knowledge in at least seven content areas; and contributing to the development of 13 peer-reviewed publications, eight professional presentations, one Masters of Education project, and one educational curriculum.

**Discussion:**

The number of individuals engaged in HPE research continues to rise. The MESS model could be adapted for use at other institutions, thereby reducing barriers HPE researchers face, providing an effective framework for trainees interested in education-focused careers, and having a broader impact on the education research landscape.

Successful production of high-quality studies that advance health professions education (HPE) through evidence-based research has become increasingly important for HPE faculty. The Accreditation Council for Graduate Medical Education (ACGME) requires graduate medical education (GME) faculty to ‘establish and maintain an environment of inquiry and scholarship with an active research component’ (1). In addition, some academic health centers have formalized education-specific tracks (2, 3), and academic medical centers and HPE faculty are seeking opportunities for wider dissemination of their local work (4). Continued advancement of high-quality HPE research is dependent upon the development of educators who can design and assess HPE research with the same rigor demanded in basic science or clinical research.

HPE scholarly activity is surging, yet manuscript acceptance rates for most desirable medical education journals have decreased to 10–15%. This decrease in acceptance rates may be partially due to the fact that many junior- to mid-career medical educators face seemingly insurmountable obstacles that preclude the high-caliber research required for publication (5, 6). Some of these barriers include lack of training, protected time or funding, competing responsibilities, small learner numbers, challenges implementing randomized controlled trials within educational settings, lack of accessible education research mentors, and difficulty in defining relevant and measurable outcomes (7). These faculty are often the mentors for graduate medical trainees who are interested in careers in medical education.

Several national resources exist to address these concerns. Specialty-specific organizations offer longitudinal programs that promote skills in medical education scholarship (8). Nationally recognized programs, such as Harvard Macy, establish faculty leaders in medical education (9). These national resources provide excellent developmental opportunities, but many carry their own barriers that, ironically, make them inaccessible to the educators who may reap the maximum benefit from them. For example, most (if not all) of these programs are limited to small numbers of faculty and require financial resources and institutional support for protected time.

With these challenges in mind, we sought to develop an effective local program that would foster a supportive environment and engage faculty across the medical education continuum who were committed to medical education as a career. Increasing their productivity and skillset in education may improve their ability to mentor trainees and develop future clinician-educators. Furthermore, we wanted to promote educational scholarship while minimizing costs and away time.

## Methods

In response to the education research and scholarship barriers we identified, educators at Duke University School of Medicine convened the Medical Education Scholarship Support (MESS) group, whose mission was to provide a collaborative peer-mentoring environment in education research and scholarship and to improve the production of high-quality scholarly outputs. Using a modified Delphi process, MESS specified six objectives, outlined in Table 1.

**Table 1:** MESS objectives and associated outcomes

Objectives	Outcomes
1. Establish a unique community of medical educators	Community established in the summer of 2013 100% participant retention
2. Acquire new learning (knowledge, skills, attitudes) related to medical education scholarship	Developed increased knowledge on the following topics: Medical education journals Categories for journal submissions Reliability Validity Quality improvement Endnote Author order Effective literature reviews Survey design
3. Increase MESS members’ scholarly output	Submissions to peer-reviewed journals Collaborative grant applications Local, regional, and national presentations Multi-institutional projects
4. Increase scholarly collaboration within MESS	Submissions to peer-reviewed journals Collaborative grant applications Local, regional, and national presentations Multi-institutional projects
5. Incorporate group feedback to enhance scholarly output	Active collaboration and discussion has contributed to a considerable increase in scholarly activities for group members
6. Share internal and external resources on an ongoing basis	Information on master-level medical education programs Advice on and resources for determining the best journals for publication on specific topics Key LIME podcasts Numerous articles from the literature Evaluation tools

Key LIME, Key Literature in Medical Education; MESS, Medical Education Scholarship Support.

Eight participants founded MESS during the summer of 2013, consciously making an effort toward diverse representation across disciplines (physicians and non-physicians); training (MD/DO, PhD, EdD, and MSLS); professional role (physicians, librarian, and educators); focus on the learner continuum (students, residents, fellows, and faculty); departments (pediatrics, medicine, pathology, GME, and undergraduate medical education); and academic rank (trainees, instructors, assistant professors, associate professors, and administrators). Intentionally, members of our MESS group also possessed complementary areas of expertise (e.g., research design and assessment, institutional review board, literature review, and manuscript editing).

We created MESS based on the principles of a community of practice (10). We convened as a group of individuals with a shared passion for medical education, effective teaching, quality education programs, and medical education scholarship. The group's design was focused on two key community of practice elements: domain and practice. Domain refers to a ‘shared competence that distinguishes members from other people’. The shared competence, in our case, was medical education scholarship. Practice refers to the way in which members ‘develop a shared repertoire of resources: experiences, stories, tools, ways of addressing recurring problems – in short, a shared practice’ (10). MESS has functioned as a shared practice since its inception.

Like so many homegrown communities of practice, MESS quickly evolved into a hybrid of what was planned and ‘what just worked’. We held regular 90-min monthly meetings, with an established meeting day and time. The group's process evolved organically, and less structure seemed to work most effectively. MESS has functioned as an adhocracy; a loose, flexible, and harmonious structural framework in which members are always considered equals (11). More specifically, we created a nurturing, circular social architecture with multiple, open, and diffuse lines of communication among members; a so-called web of inclusion (12). Rather than selecting a single leader or even having rotating leaders, we allowed members’ evolving needs to drive the group discussions, raising topics on an as-needed basis. As each meeting approached, members suggested topics for discussion via e-mail based on current education research challenges, questions we had, or feedback one of us may have needed on a work in progress.

We focused our discussions on and developed new knowledge around a variety of issues education scholars face regularly, including politics of author order; submission process for medical education institutional review board proposals; appropriate responses to journal reviewers; study methods, assessments, and analyses; and appropriate venues for dissemination of medical education research. As a practice, our meetings generally did not require preparation prior to the meeting. If the group did not have immediate solutions to issues raised in the meetings, then the group would actively research the issues in real time, discussing the collective resources (human or otherwise) that would guide them to a solution. Attendance and most importantly collaboration were always the norm. Three members who have since moved to roles at other institutions continue to actively participate in meetings via video-conferencing to maintain their ongoing involvement.

This manuscript describes a process rather than a formal investigation; therefore, institutional review board review was not applicable.

## Results

### Feasibility

The MESS group worked diligently to determine a commonly acceptable meeting time so that the majority of members would be able to attend meetings, and participation would not adversely interfere with other professional responsibilities. Each monthly meeting has been attended by at least 50% of the group members. If a greater number of members were unable to attend a given meeting, then the meeting would be cancelled ahead of time. MESS has never required financial support, protected time, or travel/conference costs.

### Outcomes

MESS has proven itself to be a sustainable unfunded homegrown local community of practice, encouraging faculty to pursue HPE careers and fostering scholarship. We have met each of our six objectives (Table 1) including maintaining 100% participant retention for over 2 years; developing increased knowledge in at least seven content areas; and contributing to the development of 13 peer-reviewed publications, eight professional presentations, one Masters of Education project, and one educational curriculum.

Participants have shared their individual knowledge and skills with each other. We have also shared feedback and provided concrete answers to questions. Importantly, participants provide each other ongoing support and encouragement, which has proven invaluable.

## Discussion

MESS is a sustainable and unfunded homegrown local community of practice that promotes faculty pursuing HPE careers and fosters scholarship by collaborating outside of their traditional peer group (e.g., GME). We have successfully met the stated objectives and demonstrated commitment to scholarship. Members have been impressed with the quality of support and impact on scholarship; many works have been collaborations with trainees and GME leadership. Although we cannot attribute all gains to MESS alone, participants report that MESS has been a valuable resource in individual and team contributions to medical education research literature. Formal regional and national education scholarship programs are excellent opportunities for faculty, and we wholly support participation. Nevertheless, for educators who are unable to attend such programs or for those seeking local support, a MESS model may be a viable alternative to foster careers in medical education and move faculty toward meeting their own development needs in education scholarship and mentorship.

Through maturation, MESS has broadened its focus and now serves as a model for the Duke Academy for Health Professions Education and Academic Development (AHEAD), which is Duke's health professions educators’ academy. We are currently: 1) developing an enhanced structure to present quarterly faculty development sessions on topics with which novice medical education researchers (including trainees) typically struggle (e.g., institutional review board, funding, collaborating, and finding time); 2) providing individual mentoring for junior medical education researchers and trainees; 3) offering pre-screening for institutional review board submission; and 4) inviting non-members to present scholarly works in progress for feedback.

MESS has successfully established a community of medical educators, including active peer collaborators, with a diversity of interests and skill sets, while maintaining a small and comfortable environment that promotes peer support and a collegial exchange of ideas. We believe that MESS's small size is key to keeping this type of working meeting manageable. Furthermore, we feel that flexibility and lack of formal agenda have been central to MESS's success and longevity.

### Limitations

MESS has been largely successful, but some challenges have occurred. First, finding a recurring meeting time that worked for all MESS members on a consistent basis proved to be difficult. To solve this problem, we administered a detailed survey assessing members’ availability in various iterations as schedules evolved. Second, three MESS members relocated to different institutions. Fortunately, we all have maintained an active membership and have continued to participate in monthly meetings remotely. This experience taught us that, while MESS originated at Duke University School of Medicine, the group did not need to be contained within a single institution. Finally, as the group progressed, we identified gaps in our collective expertise; as a result of this realization, we have successfully recruited two additional MESS members. The newest members were selected based on the same initial guiding principles used to select the founding members.

## Conclusion

The number of individuals engaged in HPE research continues to rise. The MESS model could be adapted for use at other institutions, thereby reducing barriers HPE researchers face, providing an effective framework for trainees interested in education-focused careers, and having a broader impact on the education research landscape as a whole. Table 2 provides a working list of questions and considerations for the creation of a similar MESS group; we are hopeful that these questions will be used as a template for educators who may be interested in creating their own MESS.

**Table 2:** Template for creating a local MESS group

Consider	Local plan
WHY	Would it be meaningful and important to foster medical education scholarship at your institution? Is this something that is needed – for individuals and the institution? Would a MESS work for you?
WHO	Who might benefit personally and professionally? Who would be committed to participation? Who is likely to collaborate? Who has some experiences and complementary skills in medical education to be able to contribute? How would changes to the group be determined?
WHERE	Where is a central location? Is it easy to reserve ahead of time? Does it allow for confidential communication? Would it be conducive to sharing work on a computer and hardcopies? Would it allow people to see and hear each other easily?
WHEN	When is a minimally stressful time? Should it connect with a meal? Should it be a way to start or end the week? How often should it occur? At what time of the day and for how long?
HOW	What format might work for you? Do you need formal objectives for your group? Do you need an agenda for sessions? If so, how will it be formulated? Do you need a leader? How will sessions be planned and run? Would there be any follow up?

MESS, Medical Education Scholarship Support.

## Previous Presentations

The authors have presented the MESS concept via poster presentation at the 2014 Duke AHEAD (Academy for Health Professions Education and Academic Development) Education Day and the 2015 Center for Instructional Development and Education Research (CIDER) conference. The MESS concept was accepted for presentation as an oral abstract at the 2015 AAMC Medical Education meeting.
